# Bias of complete-case analysis of williams square crossover designs when data are missing not at random

**DOI:** 10.1186/1745-6215-16-S2-P140

**Published:** 2015-11-16

**Authors:** Sofia Bazakou, Robin Henderson, Linda Sharples, John Matthews

**Affiliations:** 1 Newcastle University, Newcastle upon Tyne, UK; 2 University of Leeds, Leeds, UK

## 

A Williams square is a particular type of Latin square that is widely used for crossover trials when comparing *t* treatments over *t* treatment periods. As with most forms of clinical trials, crossover trials do not always obtain all the observations prescribed in the design and the analyst has to deal with missing data. An intention-to-treat (ITT) analysis would analyse the available observations and, if these were continuous, a linear model with random patient effects and fixed effects for period and treatment would often be used. However, for a crossover design a stronger argument might be made for analysing only complete cases than would be possible for a parallel group design. Crossovers compare all treatment in each patient and if, e.g., an observation was missing because the patient could not tolerate the administered treatment, then including a patient who could not tolerate all treatments may not be the most pertinent basis for inference.

While a complete-case analysis may be a plausible alternative to an ITT analysis, there are likely to proponents in favour of both strategies. In this work we compare the approaches, principally by working out the bias in a complete-case analysis when data are missing not at random. Attention is focussed on a four-period Williams design and the work is discussed in the context of the TOMADO trial (*Thorax*, 69, 938-945) of mandibular advancement devices for the treatment of sleep apnoea.

